# The Grafting of Hydroxyaromatic Organics within Layered Perovskites via a Microwave-Assisted Method

**DOI:** 10.3390/molecules29122888

**Published:** 2024-06-18

**Authors:** Anamika Poduval, Kirsten D. Jones, Levon A. LeBan, John B. Wiley

**Affiliations:** Department of Chemistry and Advanced Materials Research Institute, University of New Orleans, New Orleans, LA 70148, USA

**Keywords:** topochemistry, grafting, layered perovskite, organic–inorganic hybrid

## Abstract

A new series of inorganic–organic hybrid perovskite materials were prepared by microwave-assisted grafting reactions. Simple carboxylic acids, acetic acid, and propionic acid, as well as hydroxyaromatic carboxylic acids, 3,5-dihydroxy benzoic acid (DBA), 5-hydroxyisophthalic acid (HPA), 4-hydroxybenzoic acid (HBA), and 4-hydroxy-4-biphenyl carboxylic acid (HBCA), were reacted with the Dion–Jacobson double-layered perovskite, HLaNb_2_O_7,_ and its alcoxy derivatives. Grafting was found to not occur with simple carboxylic acids, while those molecules with hydroxyls were all attached to the perovskite interlayers. Reactivity of the hydroxyaromatic carboxylic acids varied with the different layered perovskite hosts where reactions with HLaNb_2_O_7_ did not occur, and those with n-propoxy-LaNb_2_O_7_ were limited; the greatest extent of reactivity was seen with n-decoxy-LaNb_2_O_7_. This is attributed to the larger interlayer spacing available for the insertion of the various hydroxyaromatic carboxylic acid compounds. The loading exhibited by the grafting species was less than that seen with well-known long-chain alkoxy grafting groups. It is expected that the width of the molecules contributes to this where, due to the benzyl groups, the interlayer volume of the grafted moieties occupies a larger horizontal fraction, therefore minimizing the loading to the below half. X-ray powder diffraction and transmission electron microscopy studies found that grafting of the n-decoxy-LaNb_2_O_7_ intermediates with the series of hydroxyaromatics resulted in a reduction in crystallinity along with a disruption of the layer structure. Raman data on the series show little variation in local structure except for HBCA, where there appears to be a lengthening of the Nb-O apical linkage and a possible reduction in the distortion of inner-layer NbO_6_ octahedra. The optical properties of the hydroxyaromatic carboxylic acid grafted perovskites were also investigated using diffuse-reflectance UV-Vis spectroscopy. The band gaps of DBA, HPA, and HBA were found to be similar to the parent (E_g_ ≈ 3.4 eV), while the HBCA was significantly less by ca. 0.6 eV. This difference is attributed to electron withdrawal from the perovskite block to the HBCA ligand, leading to a lower band gap for the HBCA compound. The methods described herein allow for the formation of a new series of inorganic–organic hybrid materials where the products are of interest as precursors to more complex architectures as well as models for band gap modification of metal oxide photocatalysts.

## 1. Introduction

Select layered inorganic compounds are especially interesting due to their ability to incorporate multiple species in the interlayer region, allowing for tunable properties and diverse applications [1]. Dion–Jacobson layered perovskites are an important set of phases in this group that typically have the composition A’[A_n−1_B_n_O_3n+1_], where A’ is a monovalent cation such as an alkali metal ion or a proton, A is an alkaline earth metal, rare earth, or main group element, B is a transition metal, and n is the number of octahedral layers [2,3]. These compounds and their derivatives are especially prolific, exhibiting a range of important properties, including electronic/protonic conductivity, superconductivity, dielectric behavior, and photocatalytic activity [4,5,6,7,8,9,10]. Further, ion-exchangeable layered Dion–Jacobson structures are effective building blocks for constructing materials with desired characteristics as they are capable of also undergoing a wide range of intercalation and exfoliation reactions as well as other topochemical processes [1,11].

Layered perovskite derivatives can be formed via ion exchange, grafting, or intercalation. Grafting of inorganic layered structures with organic molecules can result in very interesting inorganic–organic hybrid materials [12,13]. Functionalization of the interlayer space of the perovskite lamellar structures forms interstratified inorganic sheets and organic moieties, which in turn can be utilized to modify the interlayer spacing, and the properties of subsequent hybrid compounds. Grafting reactions typically utilize protonated forms of ion-exchangeable layered perovskites, where the protons are replaced with organic cations via an ion-exchange mechanism [14,15]. Interlayer organics can be replaced by other organics by treating the structure with a solution of the targeted compound. Thus, layered perovskites with a diverse range of interlayer organic groups can be synthesized. Sugahara and coworkers have been leaders in this chemistry and have reported the insertion of n-alkoxyl, n-amines, polyethers, organophosphonic acids, etc. [16,17,18,19,20].

Most of the organic modification reactions of layered perovskites performed by conventional benchtop procedures have long reaction times (days to over a week). Because of this, it can be time-consuming to test a series of reactions. Microwave irradiation as a heating method has proven to greatly expedite reactions and has been demonstrated more generally in a series of diverse applications like environmental remediation, medicine, printing, paints, thin films, agriculture, and wood treatment [21,22]. In the production of inorganic–organic layered perovskites, this approach has been quite effective and has led to increases in yield and compound purity under milder reaction conditions [15,23,24,25,26,27]. Initially reported on by Boykin and Smith and then expanded on by our group and Wang et al., microwave processing was shown to be extremely impactful for a series of grafting and ion exchange reactions. Also, Wang et al. extended the approach to include interlayer click chemistry [25], and our group showed that exfoliation and surface modification could be achieved to produce bulk quantities of perovskite nanosheets [28].

Herein we utilized microwave-assisted organic grafting reactions with the set of hydroxyaromatic carboxylic acids, 3,5-dihydroxy benzoic acid (DBA), 5-hydroxyisophthalic acid (HPA), 4-hydroxybenzoic acid (HBA), and 4-hydroxy-4-biphenyl carboxylic acid (HBCA), on HLaNb_2_O_7_, n-propoxy-LaNb_2_O_7_, and n-decoxy-LaNb_2_O_7_. Different precursors, temperatures, and reaction times were studied for optimizing the grafting reactions. Optical properties were also investigated, and it was found that while most of the series showed similar band gaps (E_g_) to the starting materials, the HBCA grafted compound exhibited a significant reduction in E_g_. The methods presented here offer a rational approach to new functional hybrids and may allow band gap modification for applications of photocatalytic interest.

## 2. Results

**Figure 1:**
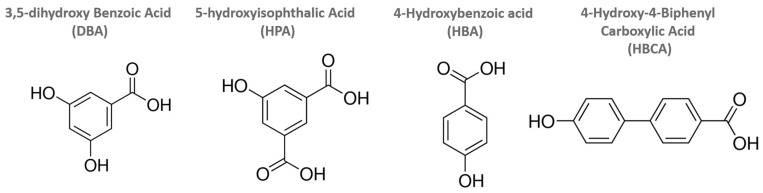
Structures of hydroxyaromatic carboxylic acids used in grafting reactions.

Microwave-assisted grafting reactions were carried out with hydroxyaromatic carboxylic acids and a set of layered perovskite hosts. All four grafting agents are aromatic with combinations of hydroxy and carboxylic acid groups (Figure 1 and Figure S1). This study examined the reactivity of the series with differing hosts, HLaNb_2_O_7,_ n-propoxy-LaNb_2_O_7,_ and n-decoxy-LaNb_2_O_7_, all derivatives of RbLaNb_2_O_7_. The reactants were systematically investigated to gauge the impact of functional groups, grafting agent dimensions, and interlayer spacing on reactivity.

It was well established by Sugahara and others [13,20] that some organics with hydroxyl groups will readily graft to the apical oxygens of layered perovskites. Carboxylic acid groups are expected to not be as reactive; we are aware of only one report of grafting involving the relatively strong acid, CF_3_COOH [29]. To verify that grafting with simple carboxylic acid groups does not occur, initially, the carboxylic acids, acetic and propionic, were examined with HLaNb_2_O_7_ and n-propoxy-LaNb_2_O_7_. No evidence for grafting was observed with any of these combinations of organic acids and perovskites (Figures S2 and S3).

Systematic reactions with the series of hydroxyaromatic carboxylic acids and layered perovskites were carried out, examining variations in reagents and microwave processing conditions. From the series of reactions conducted, it was seen that reactivity varied greatly with perovskite starting materials. The grafting reactions of the hydroxyaromatic carboxylic acids performed with HLaNb_2_O_7_ did not occur, irrespective of temperature or reaction times. This is attributed to a very small interlayer spacing; while HLaNb_2_O_7_ grafting reactions are well established with stoichiometric replacement of hydrogen by smaller organic molecules such as methanol, ethanol, and propanol [15,30], direct grafting of longer alcohols (n-pentanol and n-decanol) produce incomplete results with poor loading [15,31,32]. The proposed grafting reaction mechanism involves an initial hydrolysis followed by condensation with the loss of water [13]. For this process to take place, the grafting agent needs to infiltrate the gap between the perovskite layers [30,33]. Therefore, for larger molecules to graft effectively, greater interlayer spacing is needed [33]. n-propoxy-LaNb_2_O_7_ does have a larger layer spacing (7.7 Å versus 2.8 Å for HLaNb_2_O_7_), but only limited reactivity with this host was seen for the series. The partially reacted products showed proof in Raman spectra of grafting of the hydroxyaromatic carboxylic acid but also had bands for the remaining propoxy groups. Reactions with n-decoxy-LaNb_2_O_7_ gave the best results, readily grafting each of the hydroxyaromatic carboxylic acids.

**Figure 2:**
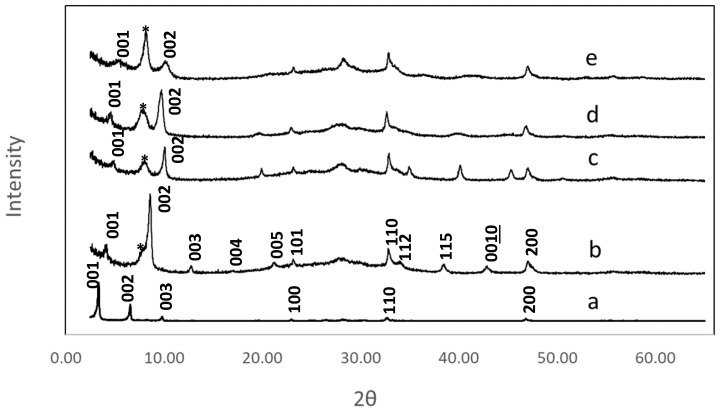
XRD patterns of n-decoxy-LaNb_2_O_7_ versus the samples after microwave reactions with hydroxyaromatic carboxylic acids, (**a**) n-decoxy-LaNb_2_O_7_, (**b**) HBCA-LaNb_2_O_7_, (**c**) HBA-LaNb_2_O_7_, (**d**) HPA-LaNb_2_O_7_, and (**e**) DBA-LaNb_2_O_7_. Select reflections are shown. The asterisk is attributed to residual HLaNb_2_O_7_ (see text).

Reactions were readily monitored by X-ray powder diffraction (XRD). Figure 2 shows powder patterns of the hydroxyaromatic carboxylic acid grafted products versus n-decoxy-LaNb_2_O_7_. In all instances, a layer contraction can be seen on reaction. Table 1 presents the change in interlayer distances relative to the parent RbLaNb_2_O_7_. Layer spacings were determined based on low angle *00ℓ* reflections. Peak broadening due to loss of crystallinity, a common feature for multistep topochemical reactions, can be seen (Figure 2). This behavior, the large unit cell, and the lack of a reliable, detailed structural model for the organization of the organic moieties in the host made reliable assignment of all reflections extremely challenging. Tenuous indexing was, however, carried out on the HBCA compound, and the assignments are shown in Figure 2b; this assumed a tetragonal unit cell where *a* = 3.85(1) and *c* = 21.04(1) Å. (The large estimated standard deviations in the cell values are consistent with the lower crystallinity.) The XRDs of the other hybrids included minimal reflections, so only the *c* parameter was estimated based on the first two *00ℓ* reflections (Table 1). Additionally, a broad impurity peak (*) is seen in each of the samples around 8.4° two-theta; this is attributed to small amounts of residual HLaNb_2_O_7_.

**Figure 3:**
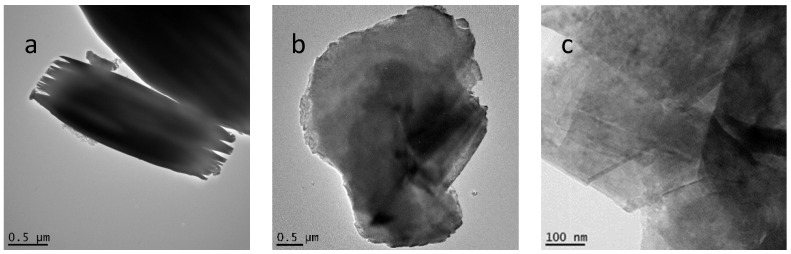
TEM images of n-decoxy-LaNb_2_O_7_ and HBCA-LaNb_2_O_7_: (**a**) n-decoxy-LaNb_2_O_7_ intermediate shows larger micro-sized crystals along with some mille-feuille-like crystallites, (**b**) HBCA-LaNb_2_O_7_ crystallites, and (**c**) HBCA-LaNb_2_O_7_ nanosheets.

Transmission electron microscopy (TEM) studies were also consistent with a dramatic reduction in crystallinity (Figure 3 and Figure S4). The intermediate, n-decoxy-LaNb_2_O_7_, contained micron and submicron-sized crystals with some exhibiting mille-feuille-like layer disruption (Figure 3a) [28]. Images for the HBCA-LaNb_2_O_7_ appear in Figure 3b,c and those for HBA-LaNb_2_O_7_, HPA-LaNb_2_O_7_, and DBA-LaNb_2_O_7_ are shown in Figure S4. All samples show degraded crystallinity and significant disruption of the perovskite layer structure. In the HBCA-LaNb_2_O_7_, clear evidence of nanosheet formation is also seen (Figure 3c).

**Figure 4:**
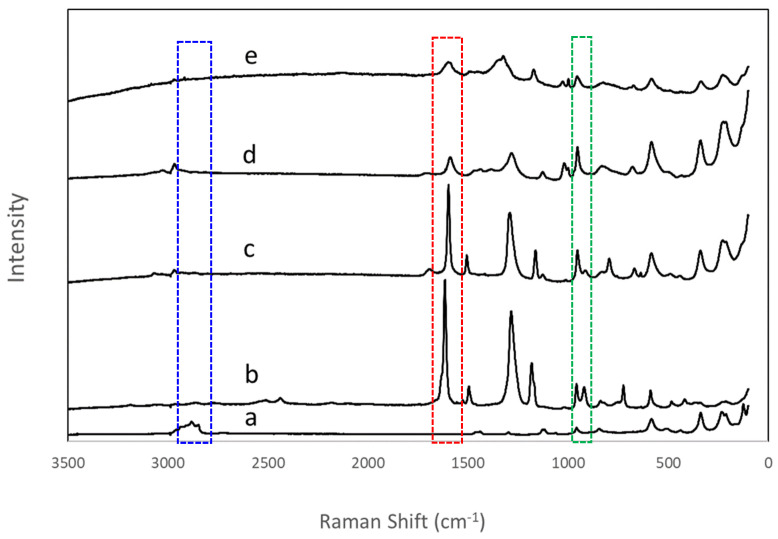
Raman spectra of the series of grafted compounds, (**a**) n-decoxy-LaNb_2_O_7_, (**b**) HBCA-LaNb_2_O_7_, (**c**) HBA-LaNb_2_O_7_, (**d**) HPA-LaNb_2_O_7_, and (**e**) DBA-LaNb_2_O_7_. The blue box (2800–3000 cm^−1^) highlights the alkyl region, the red box (~1600 cm^−1^), the aromatic region, and the green box (900–970 cm^−1^), the symmetric stretch of the apical Nb-O.

Effective replacement of the n-decoxy group by hydroxyaromatic carboxylic acids in the n-decoxy-LaNb_2_O_7_ could readily be followed by Raman spectroscopy. The appearance of the band around 1600 cm^−1^ corresponded to the addition of the aromatic group and indicated the successful introduction of all four hydroxyaromatic carboxylic acids in the interlayer (highlighted with a red dashed box in Figure 4 and Figure S5). The disappearance of the H-C-H asymmetric and symmetric stretches around 2800–3000 cm^−1^ (highlighted with a blue dashed box, Figure 4) supports the removal of the n-decoxy group. Further insight comes via consideration of the lower energy bands (<1000 cm^−1^). The band in the region 900–970 cm^−1^ is associated with the symmetric stretch (A_1g_) of the terminal Nb-O bond [34,35,36], while some bands of lower energy, 575, 480, 330, and 205 cm^−1^, appear to be associated with various vibrational modes within the perovskite blocks (Figure S6). The Nb-O symmetric stretch is known to be sensitive to local structure [34,35,36]; Table 2 presents the Raman shifts of this band for the various ALaNb_2_O_7_.

Thermal analysis of the series of grafting products allowed an estimation of the loading of hydroxyaromatic carboxylic acids into the layered perovskites. Reactions were carried out in the presence of oxygen, where decomposition of the organic components readily occurred. Representative TGA and DSC data are shown in Figure S7, and weight loss amounts are in Table S1. The loadings determined for these compounds are presented in Table 3 and are in the range of 0.39–0.49 mole grafting agent per LaNb_2_O_7_. This is notably less than what is seen in n-decoxy-LaNb_2_O_7_, which is approximately 0.9 per LaNb_2_O_7_. Also, to maintain charge balance, some additional components may be in place after displacement of the n-decoxy group; if this is H^+^ then determined loadings are a slight overestimate.

Optical studies were carried out on the series of hydroxyaromatic carboxylic acid grafted perovskites. All the samples had an off-white color except for HBCA, which was bright yellow. The diffuse reflectance spectra for DBA-LaNb_2_O_7_, HPA-LaNb_2_O_7_, and HBA-LaNb_2_O_7_ showed a reflectance edge around 400 nm (Figure S8). For the HBCA samples, the reflectance edge shifted to a higher wavelength (430–460 nm), moving to the visible blue region of absorption consistent with the prominent yellow color of the sample.

Percentage reflectance data were used to obtain Tauc plots. All the spectra were normalized to minimize the influence of the sample preparation on the optical responses. Such layered materials are reported to exhibit indirect band gaps [28,37]. The equation employed for calculating the band gap via a Tauc plot is

(ℎ𝑣𝛼)^1/𝑛^ = 𝐴 (ℎ𝑣 − E_𝑔_) (1)

where *n* values are ½ for direct band gap materials and 2 for indirect. The terms ℎ, *v*, E*_g_*, and A are Planck’s constant, frequency, band gap, and proportional constant, respectively. The calculated Kubelka–Munk function, F(*R*), can then replace the absorption coefficient (*α*):

F(𝑅) = (1 − 𝑅)^2^/2𝑅 (2)

**Figure 5:**
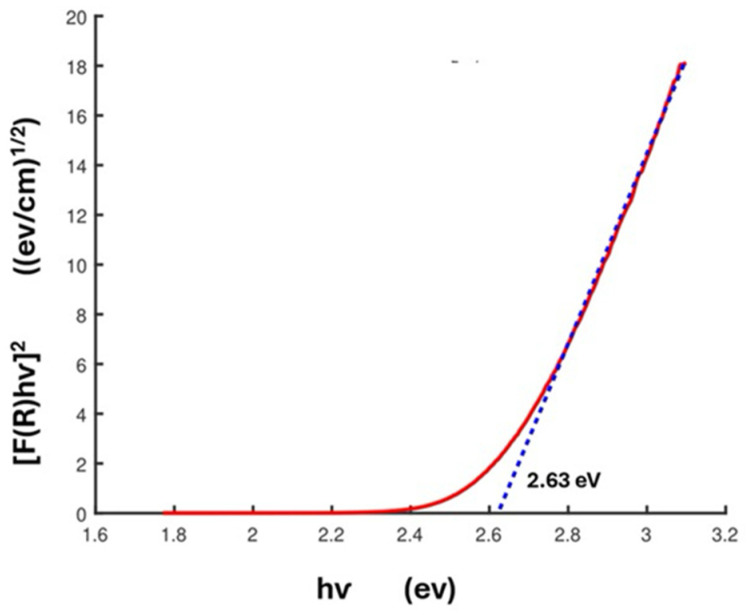
Tauc plot used to determine the optical band gap for HBCA-LaNb_2_O_7_.

The Tauc plot is obtained by plotting [F(*R*)ℎ*v*]^2^ versus ℎ*v*. A plot for HBCA-LaNb_2_O_7_ is shown in Figure 5; extrapolating the linear section of the plot to give the corresponding ℎ*v* value leads to a calculated band gap of 2.63 eV. Table 4 shows the average band gaps obtained for the series of grafted samples, and Table S2 shows those values determined for samples produced under different reaction conditions.

## 3. Discussion

A set of hydroxyaromatic carboxylic acid perovskites was prepared by a multistep topochemical reaction strategy that included ion exchange followed by a series of strategic grafting reactions. Direct grafting of each of these agents, DBA, HPA, HBA, and HBCA, was not possible. Only via sequential processing could the molecules be introduced. Equation (3) highlights the series of topochemical steps involved:

RbLaNb_2_O_7_ → HLaNb_2_O_7_ → n-propoxy-LaNb_2_O_7_ → n-decoxy-LaNb_2_O_7_ → R-LaNb_2_O_7_ (3)

where R = DBA, HPA, HBA, and HBCA. While minimal grafting was observed in some instances with n-propoxy-LaNb_2_O_7_, reactions with the n-decoxy-LaNb_2_O_7_ precursor were always successful. Sugahara and coworkers established that the interlayer distance is important for effective grafting reactions in that for longer molecules, a correspondingly larger gallery spacing is needed [33]. The interlayer distance for n-decoxy-LaNb_2_O_7_ exceeds that of each grafted compound (Table 1), and calculations of molecular dimensions of grafting agents are consistent with this trend (Table S3). Decanol, a relatively large molecule, readily exchanges directly with n-propoxy-LaNb_2_O_7_. This indicates that maybe the molecular rigidity of the series of hydroxyaromatic carboxylic acids can also influence the ease of substitution.

To optimize the loading, microwave processing was investigated at several different temperatures and reaction times, and the best conditions overall were found to be 100 °C for 2 h. The loading for the series fell in the range of 0.39–0.49 moles/LaNb_2_O_7_ and never exceeded 0.5 moles/LaNb_2_O_7_ (Table 3). Interestingly, temperatures above 100 °C led to lower loadings. In these studies, the maximum loading of the hydroxyaromatic carboxylic acid substituents, up to approximately 0.5 moles R/LaNb_2_O_7_, is less than what is seen for the various linear n-alkoxy grafting agents, which in many instances can approach 1 mole organic per LaNb_2_O_7_ [15]. It is therefore possible that the width of these molecules combined with the relatively rigid nature of the carbon backbone reduces the uptake of the grafting agents. The large width of the substituted benzyl groups exceeds the distances between nearest (~3.9 Å) and next nearest (~5.5 Å) apical oxygens, likely interfering with the attachment of additional units. Further, n-alkyl groups have significant molecular flexibility, while functionalized benzyl groups are relatively rigid due to the aromatic.

If one considers an approximate composition of 0.5 for the series of grafted agents, for charge balance to occur, likely the remaining sites are occupied by protons. While these reactions were carried out under anhydrous conditions, it is still possible that protons could arrive via small amounts of water in the solvent or from the grafting agents themselves. The Raman data for HBCA (Figure S6 and Figure 3) give some support to this in that the single band observed for RbLaNb_2_O_7_ (~930 cm^−1^) is split into two bands (954 and 920 cm^−1^). A blue shift has been associated with the attachment of a proton [34], as in HLaNb_2_O_7,_ while a red shift occurs when the symmetric Nb-O stretch is influenced by adjacent groups. In this case, it is expected that the electron-withdrawing biphenyl group leads to a lengthening of the Nb-O bond. Although not as likely, another possibility for the ~0.5 compositions might be the formation of localized oxygen vacancies via the loss of water, such as seen in the formation of LaNb_2_O_6.5_ [40]; although the exclusive formation of this phase does not appear consistent with the Raman data.

It is known that the energy of the Ti-O band in A_2_La_2_Ti_3_O_10_ (A = Na, K, or Rb) is sensitive to A cations such that a linear arrangement of A substituents along the bond Nb-O---A can reduce bond order and lead to a red shift from Na to K to Rb [35]. If one considers the corresponding behavior in the series ACa_2_Nb_3_O_10_ (A = Na, K, Rb, or Cs) and ALaNb_2_O_7_ (A = Na, K, Rb, or Cs), the vibrational energy for the terminal Nb-O does not shift as a function of cation and remains steady at about 937 cm^−1^ and 929 cm^−1^, respectively [35]. Proton exchange of the triple-layer DJ (HCa_2_Nb_3_O_10_) leads to a blue shift of this band as the proton interacts with Nb-O [34]. In consideration of our series, similarly, the Nb-O vibrational band shifts to higher energy (960 cm^−1^) on the exchange of Rb for H in ALaNb_2_O_7_ (Table 2, Figure S6). Grafting reactions to form n-decoxy-LaNb_2_O_7_, DBA-LaNb_2_O_7_, HPA-LaNb_2_O_7_, and HBA-LaNb_2_O_7_ all have a similar impact with bands ranging from 954 to 958 cm^−1^. Additionally, similar energies are seen in n-propoxy-LaNb_2_O_7_ and n-pentoxy-LaNb_2_O_7_ [15,41], while methoxy-LaNb_2_O_7_ is blue-shifted to just above that of HLaNb_2_O_7_ [41]. In contrast, HBCA-LaNb_2_O_7_ shows two peaks. The one at 954 cm^−1^ is similar to n-decoxy-LaNb_2_O_7,_ and the other grafted hydroxyaromatic carboxylic acid perovskites, while the band at 920 cm^−1^ is considerably red-shifted, indicating a weakening of the apical Nb-O bond.

Although clear assignment for lower energy Raman bands in Dion–Jacobson double-layered perovskites have not been reported, by analogy to detailed studies on triple-layered compounds [35,42] and by comparison of the parent compounds, RbLaNb_2_O_7_ and HLaNb_2_O_7_ (Figure S6), one can expect that the bands at 575, 480, 330 and 205 cm^−1^ are associated with stretches and bends within the perovskite blocks. The band at 575 cm^−1^ is observed throughout the series of samples, and it is plausible that this is due to bending modes associated with the equatorial oxygens similar to what is described for a triple-layered Ruddlesden–Popper compound [42]. While strong major low energy bands, such as 330 and 205 cm^−1^, are retained through most of the series (ALaNb_2_O_7_, A = Rb, H, n-decoxy, DBA, HPA, and HBA), the one exception is HBCA-LaNb_2_O_7_, where the intensity of this set of these bands (100–350 cm^−1^) are greatly diminished. It has been shown that for simple perovskites (ABO_3_, A = Li, Na, K), as distortions from ideal octahedra decrease (Li → K), the intensity of the corresponding low energy bands decrease and even disappear [34]. The diminishing of low energy band intensity in HBCA-LaNb_2_O_7_ may correspond to a reduction in the distortion of NbO_6_ octahedra characteristic of the double-layered Dion–Jacobson series.

Maeda and Mallouk highlighted the various factors that are important to the magnitude of the band-gap energy of layered perovskite oxides [39]. Especially pertinent to these discussions include the consideration of the M–O–M bond angle within NbO_6_ octahedral units, where the ideal 180° Nb–O–Nb angle is most favorable for p-bonding. As the angle deviates, the excited energy state becomes localized, and the band gap becomes wider. Strong interactions by interlayer species with the apical oxygen of the NbO_6_ octahedra can oppose this localization via polarization and lengthening of the short apical Nb-O bond; this decrease in NbO_6_ distortion then leads to a reduction in the band gap. Considering the band gaps of the grafted R-LaNb_2_O_7_, most of them change little relative to the parents due to interactions with the organic substituents (Table 4). The one exception is HBCA—in this case, the band gap drops by about 0.6 eV. This is a significant change indicating that even with only a ~40% loading, the biphenyl structure of HBCA readily augments the band structure. The electron-withdrawing nature of the HBCA group appears to expand the apical Nb-O bond, reducing the distortion of NbO_6_ octahedra. Evidence for this is seen where the Raman vibration at ~950 cm^−1^ is red-shifted to 920 cm^−1^. Further, the relative reduction in the intensity of the lower energy vibrations between 100 and 400 cm^−1^ is consistent with less distorted octahedra of the perovskite inner-layer [34]. These two factors should impact the band gap and are consistent with the smaller E_g_ seen for HBCA-LaNb_2_O_7_.

## 4. Experimental

### 4.1. Synthesis

*Materials.* Rb_2_CO_3_ (Alfa Aesar, Ward Hill, MA, USA, 99.8%) was used as received. Nb_2_O_5_ (Alfa Aesar, 99.9985%) and La_2_O_3_ (Alfa Aesar, 99.99%) were heated at 1000 °C in the air for 12 h to remove any impurities in the form of hydroxides and carbonates. Organic solvents like N,N-dimethyl formamide (DMF) (Sigma-Aldrich, St. Louis, MO, USA, 99+%), 1-propanol (Alfa Aesar 99+%), 1-decanol (Alfa Aesar 98+%), acetic acid (Alfa Aesar 99+%), and propionic acid (Alfa Aesar 99+%) were used without further purification. The hydroxyaromatic carboxylic acids used for grafting reactions, 3,5-dihydroxy benzoic acid (DBA) (Alfa Aesar, 98%), 5-hydroxyisophthalic acid (HPA) (Alfa Aesar, 98%), 4-Hydroxybenzoic acid (HBA) (Alfa Aesar, 98%), and 4-hydroxy-4-biphenyl carboxylic acid (HBCA) (Alfa Aesar, 99%), were also used as received.

*Synthesis of starting materials*. RbLaNb_2_O_7_ was prepared by the ceramic method in an alumina crucible. Rb_2_CO_3_, La_2_O_3_, and Nb_2_O_5_ were combined in the molar ratio of 1.3:1:2, respectively. The mixture was ground and heated at 850 °C for 12 h, reground, and then heated at 1050 °C for 24 h, followed by grinding and reheating at 1100 °C for 24 h. The product obtained was washed with distilled water to remove any excess rubidium carbonate and soluble decomposition compounds and then dried at 90 °C for several hours.

*Microwave synthesis*. The organic grafting reactions with alcohols and hydroxyaromatic carboxylic acids, as well as the acid exchange reactions (protonation), were carried out in a StartSYNTH Microwave Synthesis Labstation (Milestone Inc., Shelton CT, USA), in which the temperature, time, and power (watts) can be controlled. The reactions were executed in quartz pressure reactors (<15 bar) with a stirring bar and Weflon button (graphite-doped Teflon) on Milestone’s START rotating platform. *Caution:* Each quartz reactor vessel should be carefully inspected for glass defects before every reaction, as these defects could result in hotspots leading to explosions.

*Protonation.* Protonation of RbLaNb_2_O_7_ was carried out with 6 M HNO_3_ via a microwave-assisted reaction for 3 h at 60 °C (maximum power of 300 W) with continuous stirring. The product, HLaNb_2_O_7_, was washed with distilled water until the pH reached 7 and dried at 75 °C for 3 h. X-ray powder diffraction data for RbLaNb_2_O_7_ and HLaNb_2_O_7_ are shown in Figure S9, and the patterns are in good agreement with the literature [15].

*Grafting of alcohols.* Microwave-assisted methods were utilized for all the grafting reactions. The reaction intermediate n-propoxy-LaNb_2_O_7_ was prepared from HLaNb_2_O_7_ following the methods of Akbarian-Tefaghi et al. [15]. Typically, 0.15 g of HLaNb_2_O_7_ was reacted with 14 mL of 80% *v*/*v* aq. soln. of n-propanol for 1 h at 100 °C with the maximum power of 350 W. n-decoxy-LaNb_2_O_7_ was made in a similar fashion, although starting from n-propoxy-LaNb_2_O_7_ and n-decanol [15]. The products were washed with distilled water and acetone and then dried at 70 °C for 1 h (Figure S2). The X-ray powder diffraction data for n-propoxy-LaNb_2_O_7_ and n-decoxy-LaNb_2_O_7_ are shown relative to HLaNb_2_O_7_ (Figure S10) and are consistent with the literature [15].

*Grafting of hydroxyaromatic carboxylic acids.* The solvents were chosen by analyzing the solubility of the hydroxyaromatic carboxylic acids and the dispersibility of the starting material. The reactions were screened at different temperatures (100 °C, 120 °C, and 150 °C) at various reaction times (1 h, 1.5 h, and 2 h) in either water or DMF with different starting materials, HLaNb_2_O_7_, n-propoxy-LaNb_2_O_7_, or n-decoxy-LaNb_2_O_7_. The best overall reaction conditions for the grafting of the hydroxyaromatic carboxylic acids were those carried out with n-decoxy-LaNb_2_O_7_ at 100 °C for 2 h in DMF.

*3,5-dihydroxy benzoic acid (DBA).* The reactions were carried out by dissolving 0.350 g of DBA in 10 mL DMF and stirring with 0.025 g of n-decoxy-LaNb_2_O_7_ for 2 h at 100 °C (maximum power of 450 W).

*5-hydroxyisophthalic acid (HPA).* An amount of 0.025 g of n-decoxy-LaNb_2_O_7_ was stirred with 0.350 g of HPA dissolved in 10 mL (DMF) for 1 h/1.5 h/2 h at 100 °C (maximum power of 450 W)/120 °C (maximum power of 650 W)/150 °C (maximum power of 800 W).

*4-hydroxybenzoic acid (HBA).* An amount of 0.025 g of n-decoxy-LaNb_2_O_7_ was stirred with 0.350 g of HBA dissolved in 10 mL of DMF for 2 h at 100 °C (maximum power of 450 W).

*4-hydroxy-4-biphenyl carboxylic acid (HBCA).* For grafting of HBCA, 0.025 g of n-decoxy-LaNb_2_O_7_ was stirred with 0.350 g of HBCA dissolved in 10 mL DMF for 2 h at 100 °C (maximum power of 450 W).

Each of the various products was washed in their respective solvents, followed by acetone, and then dried at 75 °C for 3 h.

### 4.2. Characterization

X-ray powder diffraction (XRD) data were collected on a Philips X’Pert system equipped with Cu Kα radiation (λ = 1.5418 Å, 40 keV and 45 mA) and a curved graphite monochromator from 2 to 65 degrees two theta in continuous mode at 0.02°/s. Partial lattice parameter refinement was carried out using the ChekCell program [43]. Transmission electron microscopy (TEM) images were observed on a JEOL 2010 TEM (JEOL USA, Peabody, MA, USA) with an accelerating voltage of 200 kV and 110 mA beam current. Samples were prepared by creating dilute suspensions of powders dispersed in toluene via sonication for no more than 30 s. Aliquots were taken from the top, middle, and bottom of the sample suspension and drop cast on a 200-mesh carbon-coated copper grid (Ted Pella, Redding, CA, USA). Raman spectra were obtained with a Thermo-Fisher DXR dispersive Raman spectrometer (Thermo Fisher Scientific, Waltham, MA, USA) using the λ = 532 nm line with a spectral resolution of 3 cm^−1^. Thermogravimetric analysis (TGA) and differential scanning calorimetry (DSC) measurements were performed on a TA Instrument TGA-DSC SQ600 system (TA Instrument, New Castle, DE, USA) under a dilute oxygen atmosphere (ca. 50% argon). The samples were heated in alumina pans to 1000 °C at a rate of 5 °C/min. Optical measurements were conducted under ultraviolet-visible (UV-Vis) radiation on a Cary 500 UV-Vis/NIR spectrometer. The powder sample was coated on a quartz slide to form an even thick film. For studying these solid samples, a diffuse reflectance accessory (DRA) was utilized.

## 5. Conclusions

Four new aromatic grafted perovskites were prepared by microwave processing methods. Multistep topochemical pathways were found to be effective in the insertion of a series of functionalized benzyl groups, boding well for the introduction of even more complex moieties within such layered oxide hosts. Further, the potential modification of organics after attachment, such as demonstrated by Wang et al. [25], suggests that subsequently, even more intricate structures may be formed, including those where structural variations are influenced by the confines of the perovskite gallery. In the case of carboxylic acid groups, for example, one could possibly employ interlayer condensation reactions to produce anhydrides, amides, esters, etc. Strict control of these grafted organics could also allow one to tune the band gap of the perovskite host, as seen with HBCA; this could improve oxide photocatalysts by shifting absorption into the visible region. Additional considerations come from the potential development of metal coordination chemistry, such as seen in metal–organic frameworks (MOFs) built off of carboxylic acid groups [44]. Overall, the topochemical construction and subsequent modification of organic layers within a receptive perovskite host could lead to new intricate and technologically significant inorganic–organic hybrid architectures.

## Figures and Tables

**Table 1:** Unit cell parameter (*c*), ∆*c* relative to RbLaNb_2_O_7_, and interlayer spacing of the compounds.

Compound	*c* (Å)	∆*c* (Å)	Estimated Interlayer Space (Å)
RbLaNb_2_O_7_	10.9626(3) [15]	_	3.2
HLaNb_2_O_7_	10.4527(5) [15]	−0.5	2.7
n-propoxy-LaNb_2_O_7_	15.4011(5) [15]	4.4	7.6
n-decoxy-LaNb_2_O_7_	27.2380(6) [15]	16.3	19.5
DBA-LaNb_2_O_7_	17.4(2) ^a^	6.4	9.6
HPA-LaNb_2_O_7_	18.6(3) ^a^	7.6	10.8
HBA-LaNb_2_O_7_	17.7(2) ^a^	6.7	9.9
HBCA-LaNb_2_O_7_	21.04(1) ^b^	10.1	13.3

^a^ Based on 001 and 002 reflections. ^b^ Refinement contained all major reflections.

**Table 2:** Apical Nb-O band energies for the ALaNb_2_O_7_ series.

ALaNb_2_O_7_	Apical Nb-O (cm^−1^)
Rb	928
H	960
n-decoxy	958
HBCA	954, 920
HPA	954 (920)
HBA	954
DBA	954

**Table 3:** Compositional analysis of R-LaNb_2_O_7_ series.

Grafting Compound	Loading *
n-propanol	0.85 [33]
n-decanol	0.87 [33]
3,5-dihydroxy Benzoic Acid (DBA)	0.46
5-hydroxyisophthalic Acid (HPA)	0.49
4-Hydroxybenzoic acid (HBA)	0.49
4-Hydroxy-4-Biphenyl Carboxylic Acid (HBCA)	0.39

* Moles of grafted agent per LaNb_2_O_7._

**Table 4:** Optical band gaps for the series of grafted perovskites.

Compound	Eg (eV)
RbLaNb_2_O_7_	3.21 [38]
HLaNb_2_O_7_	3.23 [39]
DBA-LaNb_2_O_7_	3.42(6)
HPA-LaNb_2_O_7_	3.50(6)
HBA-LaNb_2_O_7_	3.44(5)
HBCA-LaNb_2_O_7_	2.66(8)

## Data Availability

The data presented in this study are available in the article and Supplementary Materials.

## Supplementary Materials

The following supporting information can be downloaded at: https://www.mdpi.com/article/10.3390/molecules29122888/s1. The supporting information file contains: Figure S1. Space-filling structures of hydroxyaromatic carboxylic acids. Figure S2. XRD patterns of HLaNb_2_O_7_ after treatment with acetic acid and with propionic acid. Figure S3. XRD patterns of n-propoxy-LaNb_2_O_7_ after treatment with acetic acid. Figure S4. Select TEM images of (a) HBA-LaNb_2_O_7_, (b) HPA-LaNb_2_O_7_, and (c) DBA-LaNb_2_O_7_. Figure S5. Raman spectra of (a) HBCA versus (b) HBCA-LaNb_2_O_7_. Figure S6. Comparison of Raman spectra for (a) RbLaNb_2_O_7_, (b) HLaNb_2_O_7_, (c) n-decoxy-LaNb_2_O_7_, and (d) HBCA-LaNb_2_O_7_ symmetric stretch. Figure S7. (a) TGA and (b) DSC data for thermal analysis of HBCA-LaNb_2_O_7_. Table S1. Weight losses for the R-LaNb_2_O_7_ series determined from TGA. Figure S8. Diffuse reflectance spectra of (a) HLaNb_2_O_7_, (b) HBA (c) DBA, (d) HPA, and (e) HBCA. Table S2. Band gaps of grafted samples from various reaction conditions. Table S3. The calculated lengths and widths of the starting materials and the hydroxyaromatic carboxylic acids. Figure S9. XRD pattern of HLaNb_2_O_7_ compared with RbLaNb_2_O_7_. Figure S10. XRD pattern of HLaNb_2_O_7_ compared with n-propoxy-LaNb_2_O_7_ and n-decoxy-LaNb_2_O_7_ grafted samples. Refs. [45,46] are cited in the Supplementary Materials.
