# Supplementary material for: The Grafting of Hydroxyaromatic Organics within Layered Perovskites via a Microwave-Assisted Method
Source: Molecules. 2024 Jun 18;29(12):2888. doi: 10.3390/molecules29122888 (PMC11206368; doi:10.3390/molecules29122888)
Supplement: Supplementary file 1 [file molecules-29-02888-s001.zip › molecules-2972973-supplementary.pdf]

Supporting Information

Grafting of Hydroxyaromatic Organics within Layered Perovskites via a Microwave-assisted Method

Anamika Poduval, Kirsten D. Jones, Levon A. LeBan II, and John B. Wiley*

Department of Chemistry and Advanced Materials Research Institute, University of New Orleans, New Orleans, LA 70148

FIGURES AND TABLES

**Figure S1**	Space-filling structures of hydroxyaromatic carboxylic acids	**S2**
**Figure S2**	XRD patterns of $HLaNb_2O_7$ after treatment with acetic acid and with propionic acid	**S2**
**Figure S3**	XRD patterns of n-propoxy-$LaNb_2O_7$ after treatment with acetic acid.	**S3**
**Figure S4**	Select TEM images of a) HBA-$LaNb_2O_7$, b) HPA-$LaNb_2O_7$, and c) DBA-$LaNb_2O_7$	**S3**
**Figure S5**	Raman spectra of a) HBCA versus b) HBCA-$LaNb_2O_7$.	**S4**
**Figure S6**	Comparison of Raman spectra for a) $RbLaNb_2O_7$, b) $HLaNb_2O_7$, c) n-decoxy-$LaNb_2O_7$, and d) HBCA-$LaNb_2O_7$ symmetric stretch.	**S4**
**Figure S7**	a) TGA and b) DSC data for thermal analysis of HBCA-$LaNb_2O_7$.	**S5**
**Table S1**	Weight losses for the R-$LaNb_2O_7$ series determined from TGA.	**S5**
**Figure S8**	Diffuse reflectance spectra of a) $HLaNb_2O_7$, b) HBA c) DBA, d) HPA, and e) HBCA.	**S5**
**Table S2**	Band gaps of grafted samples from various reaction conditions.	**S6**
**Table S3**	The calculated lengths and widths of the starting materials and the hydroxyaromatic carboxylic acids.	**S6**
**Figure S9**	XRD pattern of $HLaNb_2O_7$ compared with $RbLaNb_2O_7$.	**S7**
**Figure S10**	XRD pattern of $HLaNb_2O_7$ compared with n-propoxy-$LaNb_2O_7$ and n-decoxy-$LaNb_2O_7$ grafted samples.	**S7**

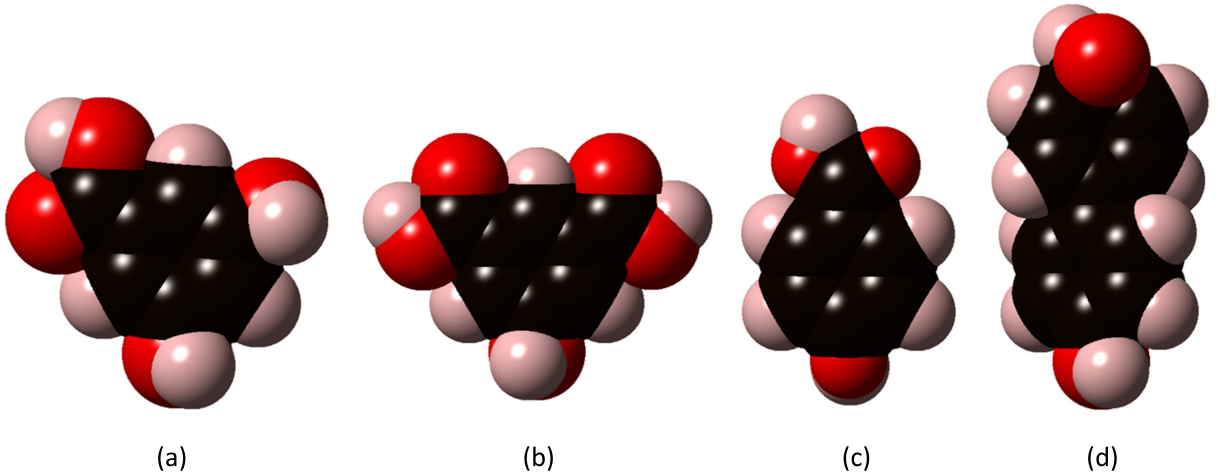

Figure S1. Space-filling structures of hydroxyaromatic carboxylic acids. (a) DBA, (b) HPA, (c) HBA, and (d) HBCA. Structures drawn with van der Waals radii of carbon (1.7 Å), hydrogen (1.2 Å) and oxygen (1.5 Å).

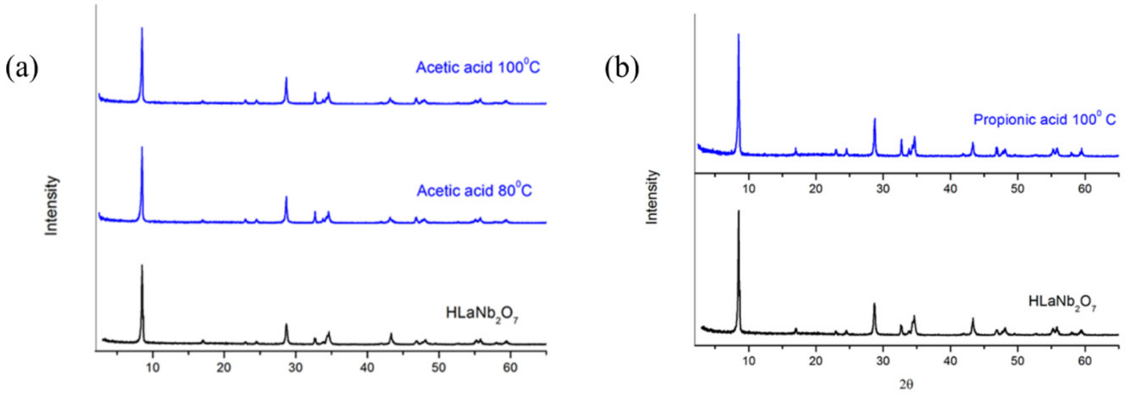

Figure S2. XRD patterns of $HLaNb_2O_7$ (a) after treatment with acetic acid and (b) after treatment with propionic acid. In both instances, no change was observed.

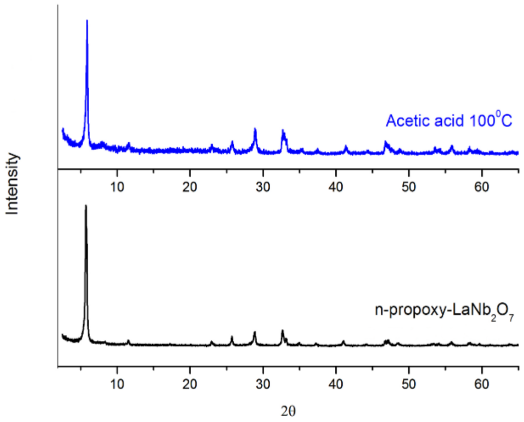

Figure S3. XRD pattern of n-propoxy-$LaNb_2O_7$ after treatment with acetic acid. No change was observed.

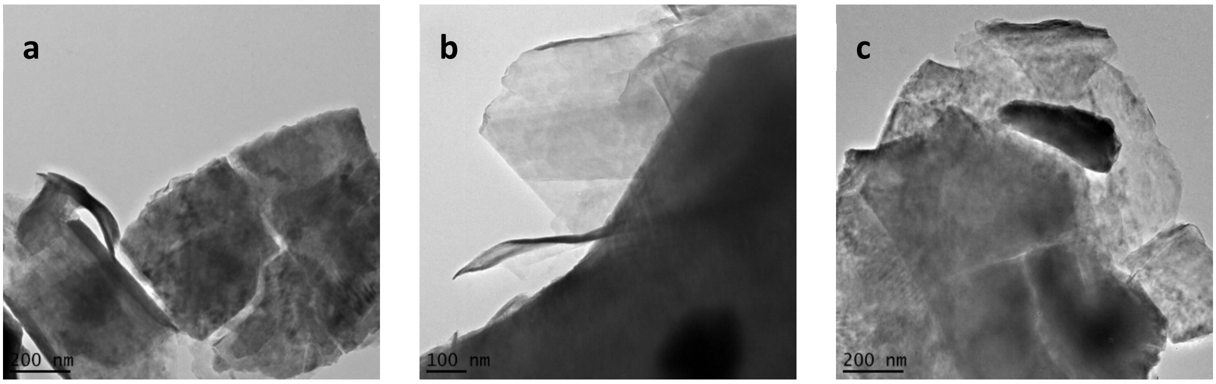

Figure S4. Select TEM images of a) HBA-$LaNb_2O_7$, b) HPA-$LaNb_2O_7$, and c) DBA-$LaNb_2O_7$. Such evidence for significant layer disruption was observed throughout the sets of samples.

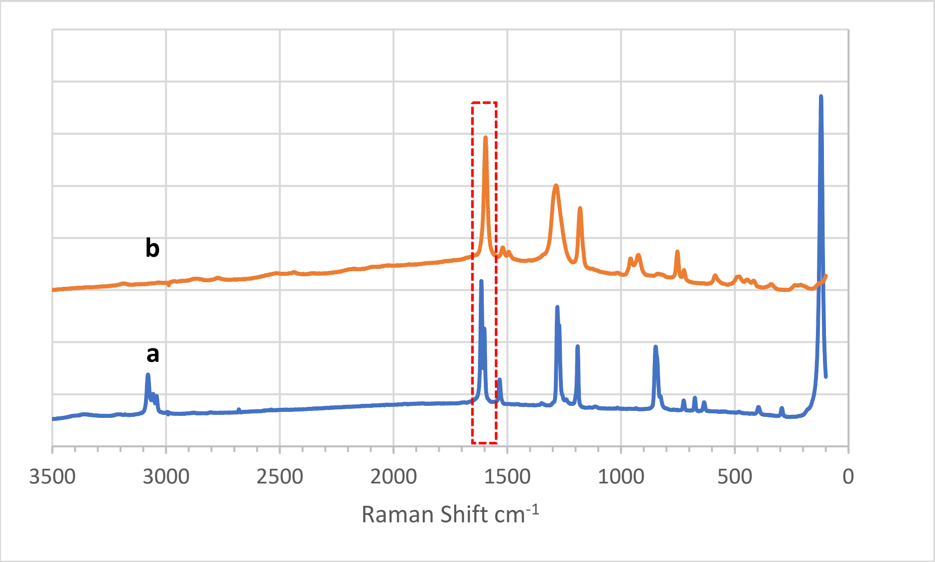

Figure S5. Raman spectra of a) HBCA versus b) HBCA-$LaNb_2O_7$. The red box highlights the aromatic region (~1600 cm^{-1}).

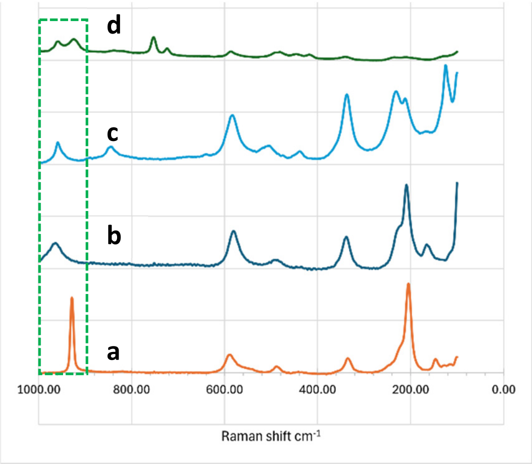

Figure S6. Comparison of Raman spectra for a) $RbLaNb_2O_7$, b) $HLaNb_2O_7$, c) n-decoxy-$LaNb_2O_7$, and d) HBCA-$LaNb_2O_7$. The green box highlights the symmetric stretch of the apical Nb-O between 900-970 cm^{-1}.

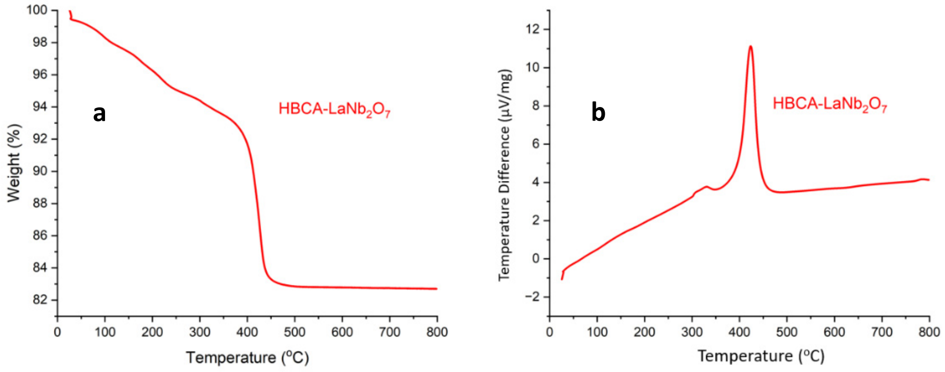

Figure S7. a) TGA and b) DSC data for thermal analysis of HBCA-$LaNb_2O_7$.

Table S1. Weight losses for the series R-$LaNb_2O_7$ determined by TGA.

Compound	Weight Loss (%)[a]
DBA-$LaNb_2O_7$	16.0(4)
HPA-$LaNb_2O_7$	15.0(2)
HBA-$LaNb_2O_7$	13.5(4)
HBCA-$LaNb_2O_7$	17.2(1)

[a]Values based on at least 3 replicates.

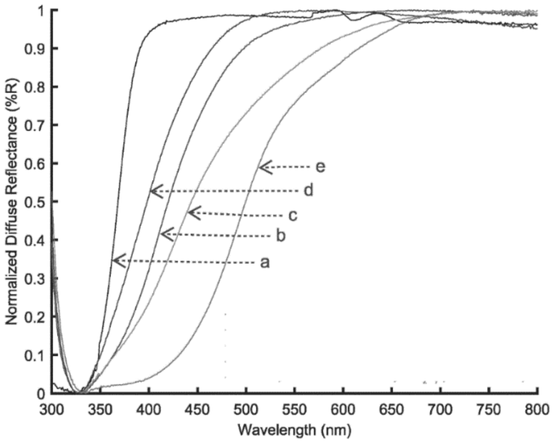

Figure S8. Diffuse reflectance spectra of a) $HLaNb_2O_7$, b) HBA, c) DBA, d) HPA, and e) HBCA.

Table S2. Band gaps of grafted samples from various reaction conditions.

Sample	Reaction conditions*			
	1h @100 °C	2h @100 °C	1h @120 °C	1h @150 °C
DBA	3.41(9)	3.42(6)	3.40(8)	3.42(10)
HPA	3.52(5)	3.50(6)	3.51(11)	3.51(9)
HBA	3.41(4)	3.44(5)	3.40(8)	3.45(7)
HBCA	2.60(6)	2.66(8)	2.70(3)	2.70(7)

*Reactions in DMF from decoxy-$LaNb_2O_7$. All reactions were run 3 times or more.

Table S3. The calculated lengths and widths of the starting materials and the hydroxyaromatic carboxylic acids.

**Compound**		**Length (Å)**	**Width (Å)**
n-propanol[a]		7.7	4.8
n-decanol[b]		17.2	4.8
3,5-dihydroxy Benzoic Acid (DBA)[a]	With one –OH connected	8.5	8.9
	With both –OH connected	9.2	7.7
5-hydroxyisophthalic Acid (HPA)[a]		8.2	10.7
4-Hydroxybenzoic acid (HBA)[a]		9.7	6.7
4-Hydroxy-4-Biphenyl Carboxylic Acid (HBCA)[a]		12.6	6.7

[a]Based on optimized structures generated in CrystalMaker® software package (Figure S1) utilizing space-filling structures with van der Waals radii (Bondi, A. "van der Waals Volumes and Radii" *J. Phys. Chem.* **1964**, *68*, 441–451).
[b]Estimate derived from consideration of n-decane crystal structure (Bond, Andrew D.; Davies, John E. "n-Decane" *Acta Crystallographica Section E*, **2002**, *58*, 196-197).

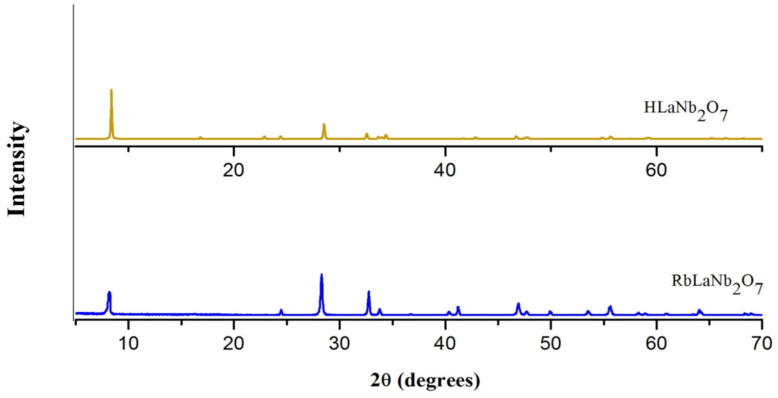

Figure S9. XRD pattern of $HLaNb_2O_7$ compared with $RbLaNb_2O_7$.

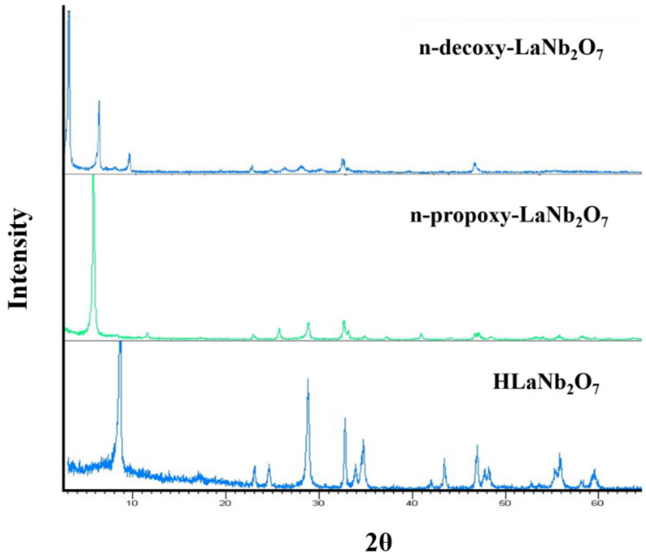

Figure S10. XRD pattern of $HLaNb_2O_7$ compared with n-propoxy-$LaNb_2O_7$ and n-decoxy-$LaNb_2O_7$ grafted samples.
